# CancerSplicingQTL: a database for genome-wide identification of splicing QTLs in human cancer

**DOI:** 10.1093/nar/gky954

**Published:** 2018-10-17

**Authors:** Jianbo Tian, Zhihua Wang, Shufang Mei, Nan Yang, Yang Yang, Juntao Ke, Ying Zhu, Yajie Gong, Danyi Zou, Xiating Peng, Xiaoyang Wang, Hao Wan, Rong Zhong, Jiang Chang, Jing Gong, Leng Han, Xiaoping Miao

**Affiliations:** 1 Key Laboratory of Environmental Health of Ministry of Education, Department of Epidemiology and Biostatistics, School of Public Health, Tongji Medical College, Huazhong University of Science and Technology, Wuhan, Hubei 430030, PR China; 2 Department of Urology, Tongji Hospital, Tongji Medical College, Huazhong University of Science and Technology, Wuhan, Hubei 430030, PR China; 3 HubeiKey Laboratory of Agricultural Bioinformatics, College of Informatics, Huazhong Agricultural University, Wuhan, Hubei 430070, PR China; 4 Department of Biochemistry and Molecular Biology, The University of Texas Health Science Center at Houston McGovern Medical School, Houston, TX 77030, USA

## Abstract

Alternative splicing (AS) is a widespread process that increases structural transcript variation and proteome diversity. Aberrant splicing patterns are frequently observed in cancer initiation, progress, prognosis and therapy. Increasing evidence has demonstrated that AS events could undergo modulation by genetic variants. The identification of splicing quantitative trait loci (sQTLs), genetic variants that affect AS events, might represent an important step toward fully understanding the contribution of genetic variants in disease development. However, no database has yet been developed to systematically analyze sQTLs across multiple cancer types. Using genotype data from The Cancer Genome Atlas and corresponding AS values calculated by TCGASpliceSeq, we developed a computational pipeline to identify sQTLs from 9 026 tumor samples in 33 cancer types. We totally identified 4 599 598 sQTLs across all cancer types. We further performed survival analyses and identified 17 072 sQTLs associated with patient overall survival times. Furthermore, using genome-wide association study (GWAS) catalog data, we identified 1 180 132 sQTLs overlapping with known GWAS linkage disequilibrium regions. Finally, we constructed a user-friendly database, CancerSplicingQTL (http://www.cancersplicingqtl-hust.com/) for users to conveniently browse, search and download data of interest. This database provides an informative sQTL resource for further characterizing the potential functional roles of SNPs that control transcript isoforms in human cancer.

## INTRODUCTION

Single nucleotide polymorphisms (SNPs) are the most frequent genetic variants in humans and represent a valuable resource for investigating the genetic basis of diseases (1). Genome-wide association studies (GWAS) have found abundant SNPs associated with various traits and diseases, but most of risk loci lack clear molecular mechanisms (2,3). Expression quantitative trait locus (eQTL) studies have been employed to identify SNPs that may influence the expression levels of genes, thereby contributing to the phenotype outcome (4–6). However, only a moderate proportion of GWAS-identified loci are strong eQTLs (7), which might be partly due to the small sample sizes, the tissues studied, and a focus on overall gene level expression measurements without consideration of transcript isoforms (8).

Alternative splicing (AS) is a molecular mechanism that produces multiple distinct transcript isoforms from a single gene. The invention of RNA sequencing greatly facilitated the identification of AS on a genomic scale (9). In human, AS can occur in ∼90% of genes in a cell type-, condition- or species-specific manner, which is thought to extensively increase the number of proteins over the number of genes in a genome (10,11). In cancer, aberrant splicing patterns are frequently observed and known to contribute to carcinogenesis, de-differentiation and metastasis (12). Many cancer-specific transcript isoforms have been identified (13). For example, an alternatively spliced transcript isoform of the gene encoding spleen tyrosine kinase is frequently expressed in breast cancer cells but never in matched normal tissues (14). Available evidence reveals that at least 20% of disease-causing single base-pair mutations affect splicing (15). Common genetic variation that affects splicing regulation, referred to as splicing quantitative trait loci (sQTLs), can lead to differences in alternative splicing between individuals, consequently influence disease susceptibility and drug response (16). Thus, the identification of sQTLs, especially in cancer tissues, might represent an important step toward fully understanding the contribution of genetic variants in tumorigenesis and development.

Because of the significance of sQTLs, several studies have performed genome-wide sQTL identifications on different human tissues, such as whole blood and brain (8,17–19). These large-scale transcriptome studies using high-throughput genotyping method and deep RNA sequencing have revealed widespread sQTLs throughout the genome. However, no database comprehensively provides sQTLs for a large number of cancer samples. To bridge this gap, we have developed a computational pipeline to systematically identify sQTLs in 33 cancer types incorporating 9026 tumor samples from The Cancer Genome Atlas (TCGA). We identified millions of sQTLs across cancer types, and constructed a user-friendly database, CancerSplicingQTL (http://www.cancersplicingqtl-hust.com/) for users to conveniently browse, search and download data of interest.

## DATA COLLECTION AND PROCESSING

### Values of splicing events collection and processing

**Figure 1.**
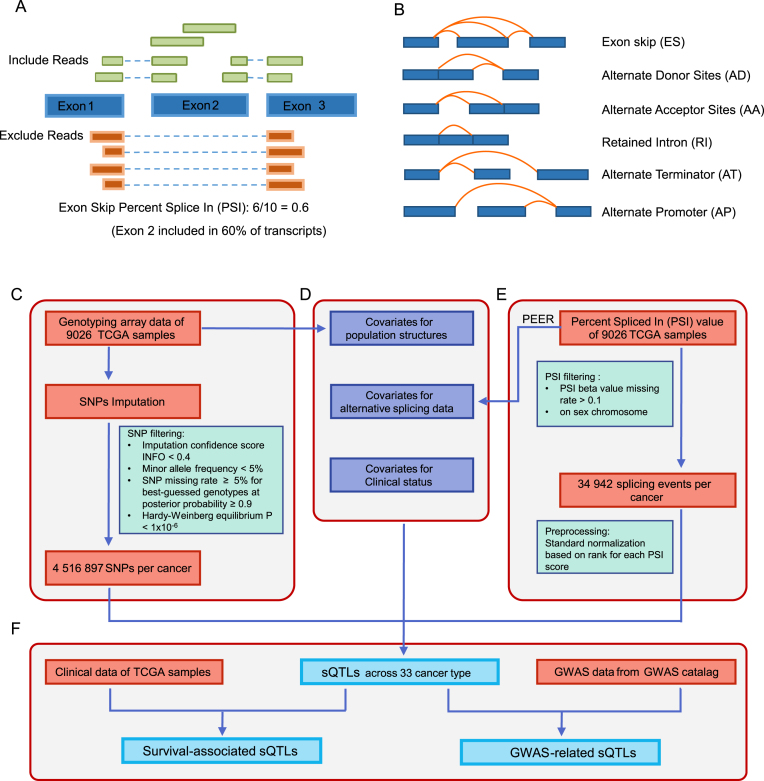
Identification of sQTLs in the CancerSplicingQTL database. (**A**) The definition of Percent Spliced In values (20). PSI is the ratio of reads indicating the presence of a transcript element versus the total reads covering the event. In this example, the PSI value is 0.6, indicating that the exon 2 is included in approximately 60% of the transcripts in the sample. (**B**) The types of splice events analyzed in SplicingQTL. (**C**) Genotype data collection and processing. (**D**) Covariates included in sQTL mapping. (**E**) The values of splice events collection and processing. (**F**) sQTLs, survival-associated sQTLs and GWAS-related sQTLs identification.

Percent Spliced In (PSI) values of each AS event were downloaded from the TCGASpliceSeq (http://projects.insilico.us.com/TCGASpliceSeq/PSIdownload.jsp) with default parameter (20). PSI value is a common, intuitive ratio for quantifying splicing events (11). The value is calculated by the transcript element present divided by the total number of reads covering the splicing event (Figure 1A). Six types of AS events were analyzed in CancerSplicingQTL, including skipped exon, retained intron, alternative donor sites, alternative acceptor sites, alternate terminator and alternate promoter (Figure 1B). For each cancer type, probes were filtered using the following criteria: (i) the rate of missing PSI value >0.1, (ii) mapping to locations on sex chromosome (Figure 1E). Finally, an average of 34 942 AS events per cancer type were used for analyses. To minimize the effects of outliers on the regression scores (21–23), the values for each probe across samples per cancer type were transformed into a standard normal distribution based on rank.

### Genotype data collection, imputation and processing

We downloaded genotype data (level 2) of 10 944 tumor samples from the TCGA data portal (https://portal.gdc.cancer.gov/), which detected the genotypes using Affymetrix SNP Array 6.0 containing 898 620 SNPs for each sample. Of these samples, 9026 samples were available with PSI data. To increase the power for sQTL discovery, we imputed autosomal variants for all samples in each cancer type using IMPUTE2, with 1000 Genomes Phase 3 as the reference panel as described in our previous study (24). To improve computation efficiency, we used the two-step procedure of IMPUTE2, which includes pre-phasing and the imputation of the phased data. Following criteria were used to exclude SNPs: (i) imputation confidence score, INFO < 0.4, (ii) minor allele frequency (MAF) < 5%, (iii) SNP missing rate ≥ 5% for best-guessed genotypes at posterior probability ≥ 0.9 and (iv) Hardy–Weinberg Equilibrium *P*-value < 1 × 10^−6^ estimated by Hardy–Weinberg R package (Figure 1C). After imputation and quality filtering, an average of 4 516 897 genotypes per cancer type were remained in the sQTL analyses.

### Covariates

In QTL analyses, covariates are often included to correct for the known and unknown confounders and increase the sensitivity of analyses (25). The top five principal components (PC) calculated by smartPCA in the EIGENSOFT program (26) were included to control for ethnicity differences, as they account for 10% of the variation explained with diminishing returns (0.5% or smaller contribution) for subsequent PCs, which are sufficient to represent the major population structure found in the TCGA dataset. Furthermore, to remove the hidden batch effects and other confounders from the AS data, we used PEER software (27) to infer hidden determinants, and selected the first 15 PEER factors from the AS data as covariates. The hidden batch effects and ethnic differences respectively accounted for an average of 19.9% and 1.19% of contribution to PSI variance in all cancers, which were described in details at the Supplementary Table S1. Other common confounders, specifically age, sex, and tumor stage, were included as additional covariates (22,28,29) (Figure 1D).

### Identification of sQTLs

For each cancer type, the effects of genetic variation on AS events were evaluated by linear regression using MatrixEQTL (30) (Figure 1F). Pairwise associations between each SNP and its splicing events around ±100 kb were calculated. The location (hg19) of splicing events was downloaded from TCGASpliceSeq database (http://projects.insilico.us.com/TCGASpliceSeq/TCGA_SpliceSeq_Gene_Structure.zip), and the SNP location (hg19) was obtained from dbSNP (https://www.ncbi.nlm.nih.gov/projects/SNP/). SNPs with false discovery rates (FDR) < 0.05 calculated by MatrixEQTL were defined as sQTLs (17).

### Identification of survival-associated sQTLs

As many AS are involved in cancer prognosis (31), sQTLs may alter gene splicing and thereby influence the prognosis. To prioritize promising sQTLs, we additionally identified sQTLs that might be associated with patient survival times. For each sQTL, we examined the associations between the sQTL and patient overall survival times. For each sQTL, samples were classified into three groups: homozygous genotype AA, heterozygous genotype Aa and homozygous genotype aa (A and a represent two alleles of one SNP). The log-rank test was used to examine the differences in survival time, and Kaplan–Meier (KM) curves were plotted to represent the survival times for each group. sQTLs with FDR < 0.05 were defined as survival-associated sQTLs.

### Identification of GWAS-associated sQTLs

The identification of causal variants is a major challenge for post GWAS studies (32). Thus we integrated the sQTLs with known GWAS risk loci to facilitate interpretation of the function of genomic variants. We downloaded all the known risk tag SNPs identified in GWAS studies from the National Human Genome Research Institute (NHGRI) GWAS catalog (http://www.ebi.ac.uk/gwas/, accessed by 1 March 2018) (2). Then we obtained GWAS linkage disequilibrium (LD) regions of these risk tag SNPs from SNAP (https://personal.broadinstitute.org/plin/snap/ldsearch.php) (33) with parameters (SNP data set: 1000 Genomes; *r*^2^ (the square of the Pearson correlation coefficient of linkage disequilibrium) threshold: 0.5; population panel: CEU (Utah Residents with Northern and Western European Ancestry), and distance limit: 100 kb). sQTLs that overlapped with GWAS tag SNPs and LD SNPs were defined as GWAS-related sQTLs.

## DATABASE CONTENT

### Samples in CancerSplicingQTL

**Figure 2.**
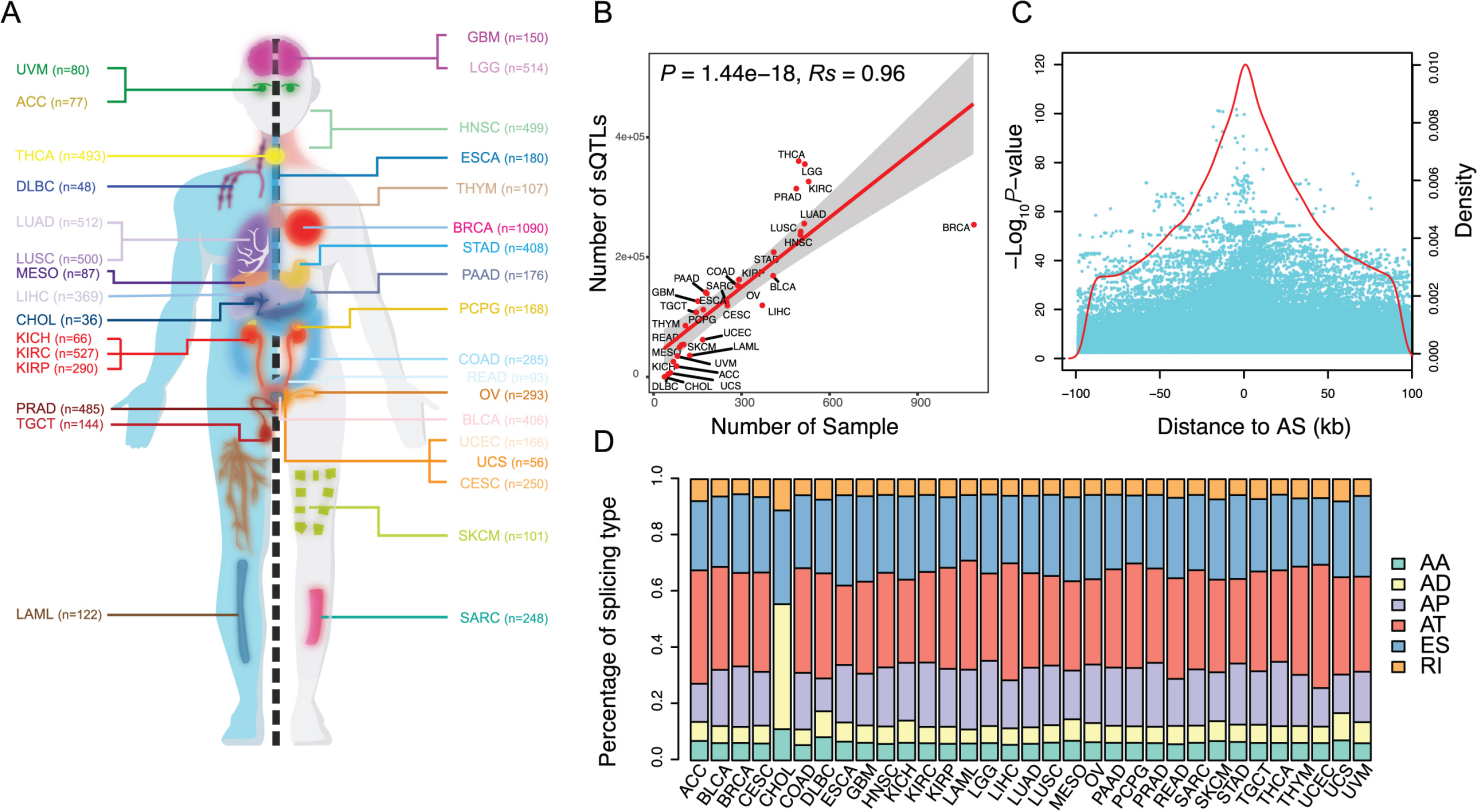
sQTL statistics. (**A**) The cancer types included in the study. (**B**) The positive correlation between the number of sQTLs and the sample size. (**C**) The distribution of sQTLs. Each cyan dot indicates a sQTL plotted according to its distance from the corresponding AS event and statistical significance of its association with AS (–log_10_*P*-value). Red line indicates density of sQTLs according to their distance from the corresponding AS event. (**D**) Bar plot indicates proportions of sQTLs affecting different AS type (AA: alternative acceptor sites, AD: alternative donor sites, AP: alternate promoter, AT: alternate terminator, ES: skipped exon and RI: retained intron).

In total, CancerSplicingQTL included 9026 tumor samples with both genotype data and PSI data available for 33 cancer types (Figure 2A). The sample size for each cancer type ranged from 36 for cholangiocarcinoma (CHOL) to 1090 for invasive breast carcinoma (BRCA) (Table 1). After imputation and quality control of the genotype data, an average of 4 516 897 SNPs per each cancer type were used for analyses, ranging from 2746 175 for BRCA to 5 120 270 for acute myeloid leukemia (LAML). After removing AS events with a rate of missing PSI beta value > 0.1 or mapping to sex chromosome, an average of 34 942 splicing events per cancer type were used for analyses, ranging from 24 707 for uterine corpus endometrial carcinoma (UCEC) to 43 937 for esophageal carcinoma (ESCA).

**Table 1.** Overview of sQTLs in each cancer type included in SplicingQTL

Cancer type	Disease full name	No. of Sample	No. of genotype	No. of splicing	sQTLs	Affected splicing	sQTL pairs	Survival_ sQTLs	GWAS_ sQTLs
ACC	Adrenocortical carcinoma	77	3567953	26620	17752	913	24950	7	4930
BLCA	Bladder urothelial carcinoma	406	4183896	32125	168597	6180	289420	157	44333
BRCA	Breast invasive carcinoma	1090	2746175	38428	253767	11961	506672	64	64008
CESC	Cervical squamous cell carcinoma and endocervical adenocarcinoma	250	4272427	33443	118989	4847	190429	412	31143
CHOL	Cholangiocarcinoma	36	4012151	31208	64	9	64	0	5
COAD	Colon adenocarcinoma	285	4491421	27466	152518	6048	255470	294	39233
DLBC	Lymphoid neoplasm diffuse large B-cell lymphoma	48	4845460	26277	4445	206	5641	0	1254
ESCA	Esophageal carcinoma	180	4463210	43937	138960	5324	214082	764	36443
GBM	Glioblastoma multiforme	150	4556997	38904	126023	4724	197274	817	33604
HNSC	Head and neck squamous cell carcinoma	499	4247759	35648	236904	8109	418356	698	60692
KICH	Kidney chromophobe	66	3771773	39171	25251	1329	34571	388	6542
KIRC	Kidney renal clear cell carcinoma	527	4579516	39696	325766	10887	600508	493	80279
KIRP	Kidney renal papillary cell carcinoma	290	4894174	33438	162228	6001	264080	1115	41681
LAML	Acute myeloid leukemia	122	5120270	29804	35478	1348	51024	152	11042
LGG	Lower grade glioma	514	4632416	41896	354837	11254	675128	1062	85201
LIHC	Liver hepatocellular carcinoma	369	4156507	26210	119209	4407	194309	229	30134
LUAD	Lung adenocarcinoma	512	4383840	37236	255517	8777	455348	147	67226
LUSC	Lung squamous cell carcinoma	500	3742393	39640	242335	9123	437645	65	62268
MESO	Mesothelioma	87	4784881	36010	49305	1734	68126	809	13856
OV	Ovarian serous cystadenocarcinoma	293	2975439	41415	149571	6769	254127	133	39361
PAAD	Pancreatic adenocarcinoma	176	4985375	39104	140937	4946	224001	771	37996
PCPG	Pheochromocytoma and Paraganglioma	168	4707250	34321	112116	4400	180122	1156	29132
PRAD	Prostate adenocarcinoma	485	4823458	37654	313993	10268	581617	1643	75506
READ	Rectum adenocarcinoma	93	4516897	29274	52896	2064	76387	204	14965
SARC	Sarcoma	248	4081096	33922	124542	4944	202118	737	33246
SKCM	Skin cutaneous melanoma	101	4865378	34942	53912	2014	74913	280	15180
STAD	Stomach adenocarcinoma	408	4306085	41433	207947	7311	338590	280	53307
TGCT	Testicular germ cell tumors	144	4791125	35758	107451	3815	166457	305	28328
THCA	Thyroid carcinoma	493	4870332	39754	359916	11265	683697	1842	86793
THYM	Thymoma	107	4892278	33234	85317	3203	132081	935	23473
UCEC	Uterine corpus endometrial carcinoma	166	4941208	24707	61884	2773	92641	372	16929
UCS	Uterine carcinosarcoma	56	3888384	32022	6586	393	8485	25	1729
UVM	Uveal melanoma	80	4737551	32067	34585	1348	47524	716	10313

### sQTLs in CancerSplicingQTL

The CancerSplicingQTL mainly contains three datasets that are sQTLs, survival-sQTLs and GWAS-sQTLs. In the sQTL analysis, the associations between each SNP and AS events within the ±100 kb window around the SNP were analyzed for sQTL mapping by linear regression. We totally identified 7 945 857 sQTL-AS pairs at a per-tissue FDR < 0.05 in 33 cancer types. In total, there are 4 599 598 sQTLs across cancer types, ranging from 64 in CHOL to 574 577 in thyroid carcinoma (THCA), with a median of 124 542 sQTLs per cancer type (Table 1). The number of sQTLs was significantly correlated with the number of samples (Spearman correlation *Rs* = 0.96, *P*-value = 1.44 × 10^−18^, Figure 2B). These sQTLs affect a median of 4847 AS events of 2857 unique genes per cancer type. Most of sQTLs were centered on AS events and 50% of sQTLs located at ±31 kb region flanking the AS events (Figure 2C). 42.8% of sQTLs were associated with multiple AS events, and of these affected AS events, 33.6%, 27.3% and 18.7% were alternate terminator (AT), exon skip (ES) and alternate promoter (AP), respectively (Figure 2D).

The germline variants derived from genotype imputation accounted for an average of 88.5% of sQTLs in all cancer types, ranging from 84.1% in BRCA to 90.1% in LAML (Supplementary Table S2). Additionally, we calculated the replication ratio of sQTL-splicing pairs in one cancer across other cancer types, finding an average of 45.9% of sQTL-splicing pairs replicate across other cancer types (Supplementary Figure S1). To compare the difference between before and after the correction of the batch effects, we respectively calculated the sQTLs between before and after the correction. We found that there were an average of 23.5% of sQTLs loss and 26.6% of sQTLs gain in all cancers, between before and after the correction of the batch effects (Supplementary Table S3).

To prioritize promising sQTLs, we linked sQTLs to patient survival times and known GWAS loci. We found 17 072 sQTLs associated with patient overall survival times across different cancer types at FDR < 0.05. The number of survival-sQTLs ranged from 0 in CHOL and lymphoid neoplasm diffuse large B-cell lymphoma (DLBC) to 1643 in prostate adenocarcinoma (PRAD). We also linked sQTL results to NHGRI GWAS Catalog data and found 1 180 132 sQTLs that overlapped with GWAS linkage disequilibrium (LD) regions of one or multiple traits.

## DATABASE ORGANIZATION AND WEB INTERFACE

CancerSplicingQTL was built based on the NodeJS 8.10.0 (https://nodejs.org/en/) framework with MongoDB 3.6.5 (https://www.mongodb.com/) as its database engine. It runs on a Linux-based Nginx Web server, while ReactJS (https://reactjs.org/), a modern JavaScript library, is used for building user interfaces. We have tested it on Google Chrome (preferred), Firefox or Apple Safari browsers. The SplicingQTL website is available online (http://www.cancersplicingqtl-hust.com/) and requires no registration.

**Figure 3.**
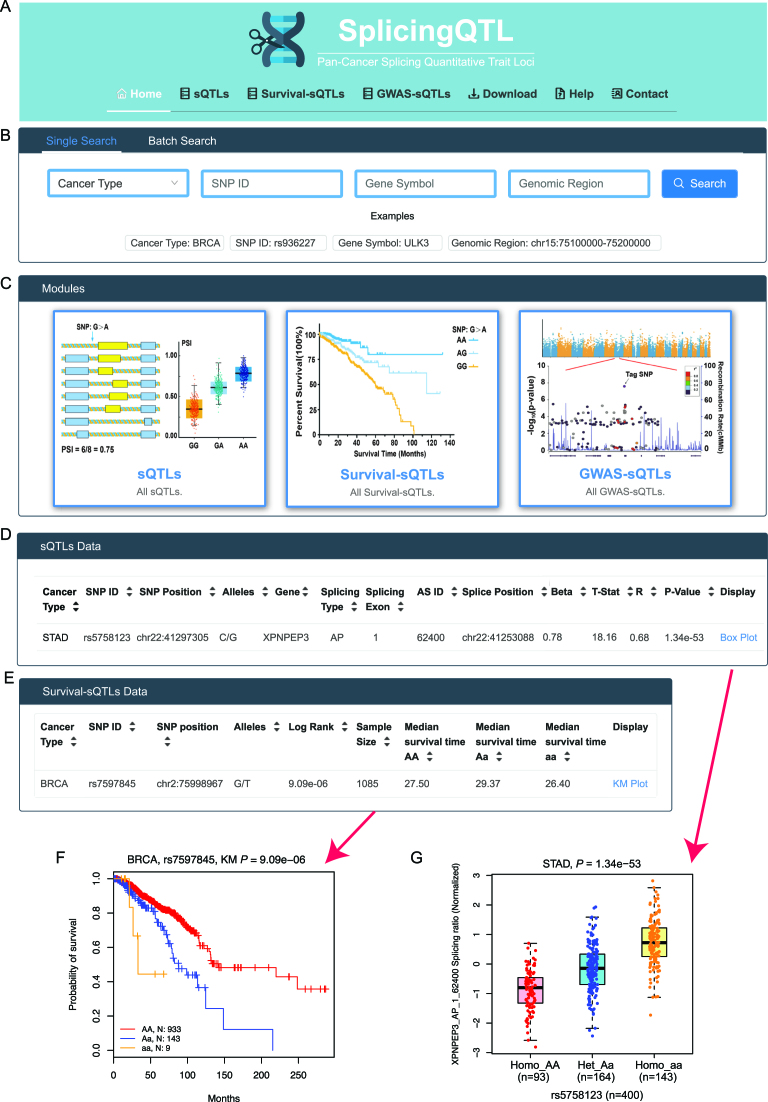
Overview of the CancerSplicingQTL database. (**A**) Browser bar in SplicingQTL. (**B**) The single and batch search boxes in SplicingQTL. (**C**) Three modules in SplicingQTL, including sQTLs, survival-associated sQTLs, and GWAS-related sQTLs. (**D**) An example of sQTL results on the ‘sQTL’ page. (**E**) An example of survival-sQTL results in ‘survival-sQTL’ page. (**F**) An example of a sQTL boxplot on the ‘sQTL’ page. (**G**) An example of a Kaplan–Meier plot on the ‘survival-sQTL’ page.

We provided a user-friendly web interface that facilitates searching, browsing and downloading the three datasets. Users can enter the ‘sQTL/survival-sQTL/GWAS-sQTL’ pages by clicking on the corresponding button in the browser bar (Figure 3A) or on hyperlinks embedded in the corresponding images in the ‘Modules’ section on the ‘home’ page (Figure 3C). Two query sections ‘Single Search’ and ‘Batch Search’ are provided for comprehensive queries across all three datasets (Figure 3B). In the ‘Single Search’ section, users can select a specific cancer type (e.g. BRCA) and input an SNP ID (e.g. rs936227), gene symbol (e.g. ULK3) or genomic region (e.g. chr15:75 100 000–75 200 000) to search sQTLs across all datasets. If users do not select cancer type, it will return results for all cancer types. The ‘Batch Search’ section allows users to input multiple cancer types, SNPs, genes or genomic regions of interest. For instance, inputting ‘rs936227’ and ‘rs9989230’ in the ‘SNP ID’ box, will return a complete list of matched entries across cancer types. In addition, a summary of the sample size, sQTL number and AS type distribution is also shown on the ‘home’ page. Putting the cursor over a cancer name on the-hand side human anatomy diagram, the matched results will show on the right-hand side figures. All data in the database can be downloaded from the ‘Download’ page. A detailed tutorial showing how the data were collected and processed is available on the ‘Help’ page. CancerSplicingQTL welcomes any feedback by email via the ‘Contact’ page.

### Query on the ‘sQTLs’ page

To query sQTLs, CancerSplicingQTL allows users to search by selecting a cancer type from a pull-down menu, or by entering a SNP ID or gene symbol. After users click the ‘Search’ button, the query results are displayed in a table showing SNP ID, SNP genomic position, SNP alleles, related gene symbol, splicing type, splicing exon, splicing ID (the same as TCGASpliceSeq annotation), splice position, beta value (effect size of SNP on PSI value), *r* value (correlation coefficient) and *P*-value of sQTL (Figure 3D). By clicking the hyperlink ‘Box Plot’ on the right of each record, a vector diagram of a boxplot will display the association between SNP genotypes and normalized PSI values. For example, our analysis showed that at *XPNPEP3* first exon, the PSI values of individuals carrying the homozygote rs5758123 aa is significantly higher than that of individuals carrying the homozygote rs5758123 AA and heterozygous rs5758123 Aa in stomach adenocarcinoma (*P*-value = 1.34 × 10^−53^, Figure 3G).

### Query on the ‘survival-sQTLs’ page

A table with SNP ID, SNP genomic position, SNP alleles, Log-rank test *P*-value, and median survival time for each genotype group is displayed on the survival-sQTLs page (Figure 3E). Search boxes are designed to retrieve specific cancer types and SNPs. If users select a specific cancer type or input a gene or SNP ID, the table will be reconstructed to display the results of the query. Each record embeds a hyperlink ‘KM Plot’, showing the association between SNP genotypes and overall survival times. For example, our analysis showed that patients with rs7597845 AA allele have a better prognosis than other patients with breast cancer (*P*-value = 9.09 × 10^−6^, Figure 3F).

### Query on the ‘GWAS-sQTLs’ page

A complete list of the SNP information, regulated splice site, related gene information and related GWAS-traits are provided on the ‘GWAS-sQTL’ page. Search boxes are designed to retrieve a specific cancer type, phenotype or SNP. In addition, users can select a different LD threshold from the ‘LD’ dropdown box to prioritize SNPs. For example, the GWAS-catalog has collected 263 tag SNPs of breast cancer risk loci. We found that 83 tag SNPs have 1402 sQTLs in their LD regions (*r*^2^ ≥ 0.5) affecting the splicing events of 100 genes. Causal variants of breast cancer could be existed among these sQTLs.

## SUMMARY AND FUTURE DIRECTIONS

In summary, CancerSplicingQTL is a comprehensive sQTL resource that uses large cancer samples to evaluate the effects of genetic variants on gene splicing. It provides a user-friendly interface for users to query, browse, and download sQTLs. To the best of our knowledge, CancerSplicingQTL is the first public database focusing on cancer-specific sQTLs. Millions of vector diagrams of sQTL box plots and KM plots are provided for scientific usage. We also identified numerous sQTLs associated with patient survival times or located in known GWAS loci that will be promising candidates for genetic research. Biologists can download entire datasets for further integrative studies.

Cancer genomics studies are developing rapidly (34,35), and we expect the number of cancer samples with genotype and splicing profiles to increase dramatically. In the future, we will continue to update CancerSplicingQTL to include more cancer samples and maintain it as a useful resource for the research community. We will add more genetic and splicing information into the database. We believe that CancerSplicingQTL will be an important resource for human cancer genetics, providing opportunities to bridge the knowledge gap from variants in sequence to phenotypes.

## Supplementary Material

Supplementary Data
