# Supplementary material for: CancerSplicingQTL: a database for genome-wide identification of splicing QTLs in human cancer
Source: Nucleic Acids Res. 2018 Oct 17;47(Database issue):D909–16. doi: 10.1093/nar/gky954 (PMC6324030; doi:10.1093/nar/gky954)
Supplement: Supplementary Data [file gky954_supplemental_files.docx]

| Supplementary Table S1. The contribution of covariates to PSI across cancer types | | | | | | |
| --- | --- | --- | --- | --- | --- | --- |
| Cancer type | All covariates | Age | Sex | Tumor stage | Ethnic group | Peer factors |
| ACC | 0.2514 | 0.0095 | 0.0018 | 0.0071 | 0.0000 | 0.2506 |
| BLCA | 0.1781 | 0.0034 | 0.0002 | 0.0025 | 0.0109 | 0.1751 |
| BRCA | 0.2079 | 0.0017 | 0.0004 | 0.0004 | 0.0139 | 0.2049 |
| CESC | 0.1710 | 0.0017 | 0.0000 | 0.0012 | 0.0044 | 0.1638 |
| CHOL | 0.1194 | 0.0081 | 0.0000 | 0.0009 | 0.0000 | 0.1107 |
| COAD | 0.1888 | 0.0044 | 0.0002 | 0.0038 | 0.0198 | 0.1849 |
| DLBC | 0.1932 | 0.0093 | 0.0000 | 0.0076 | 0.0874 | 0.2210 |
| ESCA | 0.1997 | 0.0048 | 0.0000 | 0.0000 | 0.0162 | 0.1978 |
| GBM | 0.2000 | 0.0009 | 0.0005 | 0.0000 | 0.0076 | 0.2070 |
| HNSC | 0.2024 | 0.0016 | 0.0017 | 0.0014 | 0.0128 | 0.1978 |
| KICH | 0.2160 | 0.0042 | 0.0000 | 0.0040 | 0.0000 | 0.2059 |
| KIRC | 0.2471 | 0.0054 | 0.0014 | 0.0053 | 0.0077 | 0.2394 |
| KIRP | 0.2020 | 0.0071 | 0.0033 | 0.0052 | 0.0054 | 0.1928 |
| LAML | 0.2147 | 0.0006 | 0.0000 | 0.0000 | 0.0030 | 0.2094 |
| LGG | 0.2126 | 0.0038 | 0.0005 | 0.0000 | 0.0101 | 0.2056 |
| LIHC | 0.1982 | 0.0024 | 0.0028 | 0.0015 | 0.0112 | 0.1871 |
| LUAD | 0.1796 | 0.0047 | 0.0006 | 0.0026 | 0.0149 | 0.1811 |
| LUSC | 0.1503 | 0.0011 | 0.0005 | 0.0004 | 0.0267 | 0.1452 |
| MESO | 0.1592 | 0.0000 | 0.0053 | 0.0000 | 0.0116 | 0.1752 |
| OV | 0.1763 | 0.0030 | 0.0000 | 0.0015 | 0.0088 | 0.1729 |
| PAAD | 0.2269 | 0.0032 | 0.0003 | 0.0025 | 0.0021 | 0.2307 |
| PCPG | 0.2356 | 0.0007 | 0.0000 | 0.0000 | 0.0184 | 0.2338 |
| PRAD | 0.1855 | 0.0006 | 0.0000 | 0.0000 | 0.0095 | 0.1817 |
| READ | 0.2200 | 0.0043 | 0.0000 | 0.0018 | 0.0229 | 0.2070 |
| SARC | 0.2189 | 0.0043 | 0.0024 | 0.0000 | 0.0045 | 0.2139 |
| SKCM | 0.2555 | 0.0040 | 0.0000 | 0.0036 | 0.0056 | 0.2493 |
| STAD | 0.1428 | 0.0026 | 0.0003 | 0.0008 | 0.0185 | 0.1389 |
| TGCT | 0.2963 | 0.0070 | 0.0000 | 0.0054 | 0.0057 | 0.2909 |
| THCA | 0.1698 | 0.0023 | 0.0009 | 0.0011 | 0.0039 | 0.1652 |
| THYM | 0.2924 | 0.0163 | 0.0024 | 0.0041 | 0.0000 | 0.2896 |
| UCEC | 0.2249 | 0.0017 | 0.0000 | 0.0015 | 0.0037 | 0.2169 |
| UCS | 0.1352 | 0.0036 | 0.0000 | 0.0029 | 0.0000 | 0.1349 |
| UVM | 0.2037 | 0.0024 | 0.0000 | 0.0000 | 0.0239 | 0.2002 |

| Supplementary Table S2. Statistics of sQTLs across cancer types | | | | | |
| --- | --- | --- | --- | --- | --- |
| Cancer type | Disease full name | No.of sQTL | No.of sQTL from SNP Assay | No.of sQTL from SNP imputation | Ratio of sQTL from SNP imputation |
| ACC | Adrenocortical carcinoma | 17752 | 2085 | 15667 | 88.3% |
| BLCA | Bladder urothelial carcinoma | 168597 | 19642 | 148955 | 88.3% |
| BRCA | Breast invasive carcinoma | 253767 | 40335 | 213432 | 84.1% |
| CESC | Cervical squamous cell carcinoma and endocervical adenocarcinoma | 118989 | 13939 | 105050 | 88.3% |
| CHOL | Cholangiocarcinoma | 64 | 10 | 54 | 84.4% |
| COAD | Colon adenocarcinoma | 152518 | 18089 | 134429 | 88.1% |
| DLBC | Lymphoid neoplasm diffuse large B-cell lymphoma | 4445 | 455 | 3990 | 89.8% |
| ESCA | Esophageal carcinoma | 138960 | 15529 | 123431 | 88.8% |
| GBM | Glioblastoma multiforme | 126023 | 13734 | 112289 | 89.1% |
| HNSC | Head and neck squamous cell carcinoma | 236904 | 28197 | 208707 | 88.1% |
| KICH | Kidney chromophobe | 25251 | 2776 | 22475 | 89.0% |
| KIRC | Kidney renal clear cell carcinoma | 325766 | 38460 | 287306 | 88.2% |
| KIRP | Kidney renal papillary cell carcinoma | 162228 | 17584 | 144644 | 89.2% |
| LAML | Acute myeloid leukemia | 35478 | 3500 | 31978 | 90.1% |
| LGG | Lower grade glioma | 354837 | 41044 | 313793 | 88.4% |
| LIHC | Liver hepatocellular carcinoma | 119209 | 13837 | 105372 | 88.4% |
| LUAD | Lung adenocarcinoma | 255517 | 30733 | 224784 | 88.0% |
| LUSC | Lung squamous cell carcinoma | 242335 | 31334 | 211001 | 87.1% |
| MESO | Mesothelioma | 49305 | 4994 | 44311 | 89.9% |
| OV | Ovarian serous cystadenocarcinoma | 149571 | 19389 | 130182 | 87.0% |
| PAAD | Pancreatic adenocarcinoma | 140937 | 14839 | 126098 | 89.5% |
| PCPG | Pheochromocytoma and Paraganglioma | 112116 | 12321 | 99795 | 89.0% |
| PRAD | Prostate adenocarcinoma | 313993 | 35042 | 278951 | 88.8% |
| READ | Rectum adenocarcinoma | 52896 | 5947 | 46949 | 88.8% |
| SARC | Sarcoma | 124542 | 14774 | 109768 | 88.1% |
| SKCM | Skin cutaneous melanoma | 53912 | 5440 | 48472 | 89.9% |
| STAD | Stomach adenocarcinoma | 207947 | 24305 | 183642 | 88.3% |
| TGCT | Testicular germ cell tumors | 107451 | 11155 | 96296 | 89.6% |
| THCA | Thyroid carcinoma | 359916 | 40570 | 319346 | 88.7% |
| THYM | Thymoma | 85317 | 8750 | 76567 | 89.7% |
| UCEC | Uterine corpus endometrial carcinoma | 61884 | 6654 | 55230 | 89.2% |
| UCS | Uterine carcinosarcoma | 6586 | 696 | 5890 | 89.4% |
| UVM | Uveal melanoma | 34585 | 3495 | 31090 | 89.9% |

| Supplementary Table S3. The differences of sQTLs number across cancer types between before and after the correction or control of the batch effects | | | | | | | |
| --- | --- | --- | --- | --- | --- | --- | --- |
| Cancer type | Not adjusted | Adjusted | Overlap | Overlap_not adjusted (%) | Overlap_ adjusted (%) | Loss (%) | Gain (%) |
| BLCA | 270791 | 289420 | 223549 | 82.6% | 77.2% | 17.4% | 24.3% |
| BRCA | 507902 | 506672 | 396456 | 78.1% | 78.2% | 21.9% | 21.7% |
| CESC | 182062 | 190429 | 149139 | 81.9% | 78.3% | 18.1% | 22.7% |
| CHOL | 110 | 64 | 57 | 58.2% | 89.1% | 48.2% | 6.4% |
| COAD | 260386 | 255470 | 192447 | 73.9% | 75.3% | 26.1% | 24.2% |
| DLBC | 8125 | 5641 | 4631 | 57.0% | 82.1% | 43.0% | 12.4% |
| ESCA | 224753 | 214082 | 159617 | 71.0% | 74.6% | 29.0% | 24.2% |
| GBM | 178172 | 197274 | 142258 | 79.8% | 72.1% | 20.2% | 30.9% |
| HNSC | 366502 | 418356 | 322524 | 88.0% | 77.1% | 12.0% | 26.1% |
| KICH | 42846 | 34571 | 24813 | 57.9% | 71.8% | 42.1% | 22.8% |
| KIRC | 505890 | 600508 | 433258 | 85.6% | 72.1% | 14.4% | 33.1% |
| KIRP | 234432 | 264080 | 195654 | 83.5% | 74.1% | 16.5% | 29.2% |
| LAML | 44649 | 51024 | 33004 | 73.9% | 64.7% | 26.1% | 40.4% |
| LGG | 570097 | 675128 | 509167 | 89.3% | 75.4% | 10.7% | 29.1% |
| LIHC | 198324 | 194309 | 148633 | 74.9% | 76.5% | 25.1% | 23.0% |
| LUAD | 402272 | 455348 | 351221 | 87.3% | 77.1% | 12.7% | 25.9% |
| LUSC | 397224 | 437645 | 354030 | 89.1% | 80.9% | 10.9% | 21.0% |
| MESO | 68323 | 68126 | 51351 | 75.2% | 75.4% | 24.8% | 24.6% |
| OV | 228653 | 254127 | 195793 | 85.6% | 77.0% | 14.4% | 25.5% |
| PAAD | 187507 | 224001 | 152233 | 81.2% | 68.0% | 18.8% | 38.3% |
| PCPG | 148858 | 180122 | 123207 | 82.8% | 68.4% | 17.2% | 38.2% |
| PRAD | 518120 | 581617 | 453588 | 87.5% | 78.0% | 12.5% | 24.7% |
| READ | 82879 | 76387 | 57928 | 69.9% | 75.8% | 30.1% | 22.3% |
| SARC | 170501 | 202118 | 143727 | 84.3% | 71.1% | 15.7% | 34.2% |
| SKCM | 72687 | 74913 | 52768 | 72.6% | 70.4% | 27.4% | 30.5% |
| STAD | 335281 | 338590 | 267469 | 79.8% | 79.0% | 20.2% | 21.2% |
| TGCT | 134567 | 166457 | 105670 | 78.5% | 63.5% | 21.5% | 45.2% |
| THCA | 623696 | 683697 | 537912 | 86.2% | 78.7% | 13.8% | 23.4% |
| THYM | 113315 | 132081 | 84276 | 74.4% | 63.8% | 25.6% | 42.2% |
| UCEC | 87361 | 92641 | 65879 | 75.4% | 71.1% | 24.6% | 30.6% |
| UCS | 16693 | 8485 | 6912 | 41.4% | 81.5% | 58.6% | 9.4% |
| UVM | 53733 | 47524 | 35550 | 66.2% | 74.8% | 33.8% | 22.3% |


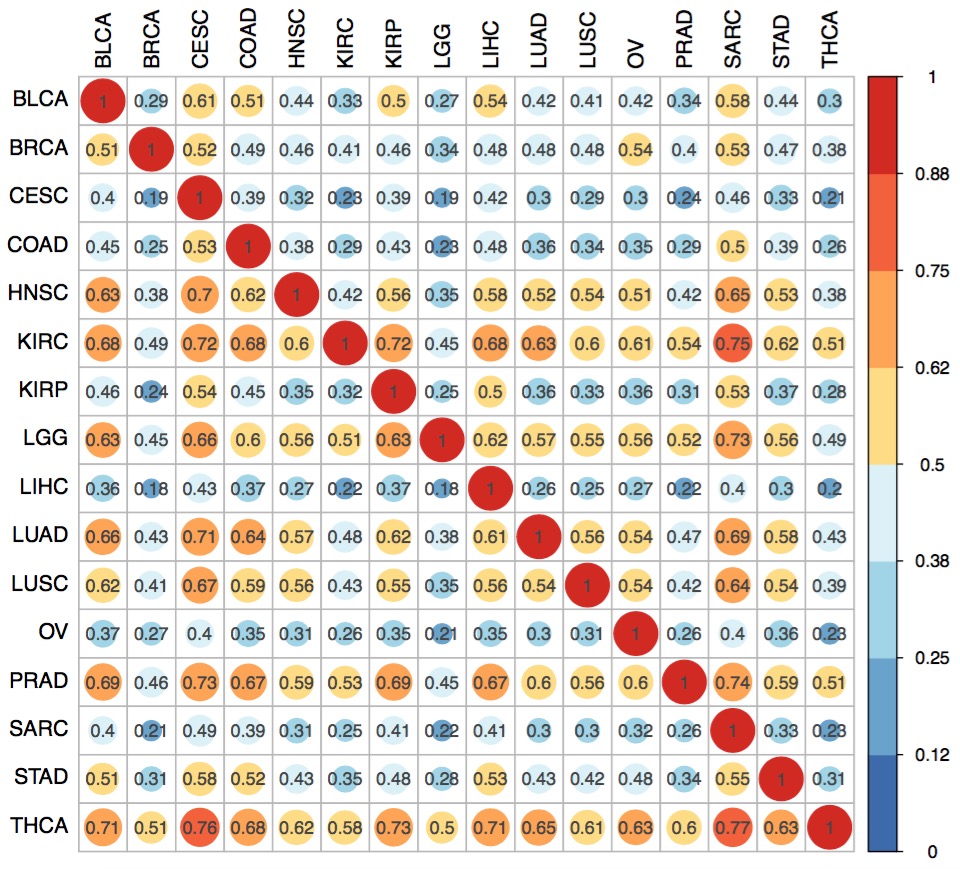


**Supplementary Figure S1**. Replicability of sQTL-splicing pairs across cancer types. The value in each circle represents the replication ratio of sQTL-splicing pairs of one cancer (y-axis) in another cancer type (x-axis).
